# Safety and efficacy of re-treatment with [^177^Lu]Lu-DOTA-Octreotate radionuclide therapy in progressive gastro-entero-pancreatic neuroendocrine tumours – a single centre experience

**DOI:** 10.1007/s00259-025-07235-w

**Published:** 2025-03-26

**Authors:** Raghava Kashyap, Ramin Alipour, Emma Boehm, Kerry Jewell, Aravind S. RaviKumar, Anthony Cardin, Javad Saghebi, Michael S. Hofman, Michael T. Fahey, Michael Michael, Tim Akhurst, Rodney J. Hicks, Grace Kong

**Affiliations:** 1 Department of Nuclear Medicine, Peter MacCallum Cancer Centre, Melbourne, Australia; 2 Sir Peter MacCallum Department of Oncology, University of Melbourne, Melbourne, VIC Australia; 3 Centre for Biostatistics and Clinical Trials, Peter MacCallum Cancer Centre, Melbourne, VIC Australia; 4 Department of Medical Oncology, Peter MacCallum Cancer Centre, Melbourne, Australia; 5 Melbourne Theranostic Innovation Centre, Melbourne, Australia; 6 Department of Medicine, St Vincent’s Hospital, The University of Melbourne, Melbourne, VIC Australia; 7 Department of Medicine, Central Clinical School, The Alfred Hospital, Monash University, Melbourne, VIC Australia; 8 Department of Clinical Pathology, Collaborative Centre for Genomic Cancer Medicine, University of Melbourne, Melbourne, VIC Australia; 9 Peter MacCallum Cancer Centre, 305 Grattan Street, Melbourne, 3000 Australia

**Keywords:** Gastroenteropancreatic (GEP), Neuroendocrine neoplasms (NENs), PRRTLutetium-177, DOTATATERe-Treatment

## Abstract

**Aim:**

Patients with gastro-entero-pancreatic neuroendocrine tumours (GEP NET) who retain somatostatin receptor (SSTR) expression after initial response to [^177^Lu]Lu-DOTA-Octreotate (LuTate) peptide receptor radionuclide therapy (PRRT) are amenable to re-treatment (R-PRRT) upon progression. We assessed the safety and efficacy of R-PRRT in patients with progressive metastatic GEP NET.

**Materials and methods:**

A retrospective analysis, approved by institutional ethics board, was performed in patients with GEP NET who received R-PRRT for either symptomatically or radiologically progressive disease. Safety was assessed by renal and haematological parameters at 3 months post R-PRRT (CTCAE v5.0). Molecular imaging response was evaluated on [^68^Ga]Ga-DOTA-Octreotate (GaTate) PET/CT using pre-defined criteria. RECIST 1.1 responses 3 months post R-PRRT were documented when feasible. Progression-free and overall survival analysis were performed.

**Results:**

A total of 63 patients had R1-PRRT (1–3 cycles). The majority (70%) had Grade 2 NET and small intestinal primary (51%). A second re-treatment course (R2-PRRT) was given in 20 patients and a third course (R3-PRRT) in 6 patients. Glomerular filtration rate (GFR) was stable following R1-PRRT. Following R2-PRRT, worsening GFR from CTCAE G2 to G3 was seen in 10% (2/20) of patients, but none after R3-PRRT. Grade 3 thrombocytopenia occurred in 2 patients after R1-PRRT and in 1 patient after R3-PRRT. Grade 4 thrombocytopenia was observed in 1 patient post R1-PRRT. Following R1-PRRT, RECIST 1.1 responses CR, PR, SD was 0%, 10%, 76%, respectively. Disease control rate on GaTate PET/CT was 52/58 (89%) post R1-PRRT. Median progression free survival (PFS) following R1-PRRT was 1.6 years (95% CI:1.2–2.3).

**Conclusion:**

R-PRRT is feasible, tolerable and efficacious in achieving disease control in patients with progressive GEP NET.

**Supplementary Information:**

The online version contains supplementary material available at 10.1007/s00259-025-07235-w.

## Introduction

A recognised characteristic of well-differentiated neuroendocrine tumours (NET) is the high expression of somatostatin receptor (SSTR) on the cell surface [1]. This feature enables radiolabelled somatostatin analogues for imaging and diagnosis [2–4] and the use of peptide receptor radionuclide therapy (PRRT) for treatment of NET [5, 6]. The NETTER-1 study [7] has shown longer progression free survival and improvement in quality of life [8] in patients treated with [^177^Lu]Lu-DOTA-Octreotate (LuTate), (LUTATHERA^®^, Novartis) PRRT compared to high-dose somatostatin analogue in well differentiated small intestinal NET. It is also shown recently from the NETTER-2 study [9] that LuTate therapy demonstrates meaningful objective response and delays progression in grade 2 and 3 NET, including pancreatic NET. The consensus guidelines [10] and the accepted practice is to administer 4 cycles of LuTate for gastro-entero-pancreatic (GEP) NET. However, despite favourable responses to the 4 cycles of initial LuTate (I-PRRT), disease eventually progresses or recurs in many patients.

In this context, further re-treatment with LuTate (R-PRRT) may be a potential option if all sites of disease retain high SSTR expression. There is increasing data on the feasibility and effectiveness of R-PRRT based on retrospective series [11–20]. Due to the relatively low incidence of the disease, some of the available studies have grouped conditions such as bronchial NET, Pheochromocytoma/Paraganglioma along with GEP NET in their analysis. The isotope and route of administration of therapy also varied among the studies. The majority of available series involved small numbers and a single retreatment cycle.

The aim of this study is to evaluate the effectiveness and safety of R-PRRT in patients with progressive metastatic GEP NET after a period of initial response to I-PRRT, and to explore potential predictive factors for response, from a European Neuroendocrine Tumor Society (ENETS) Centre of Excellence.

## Materials and methods

**Fig. 1:**
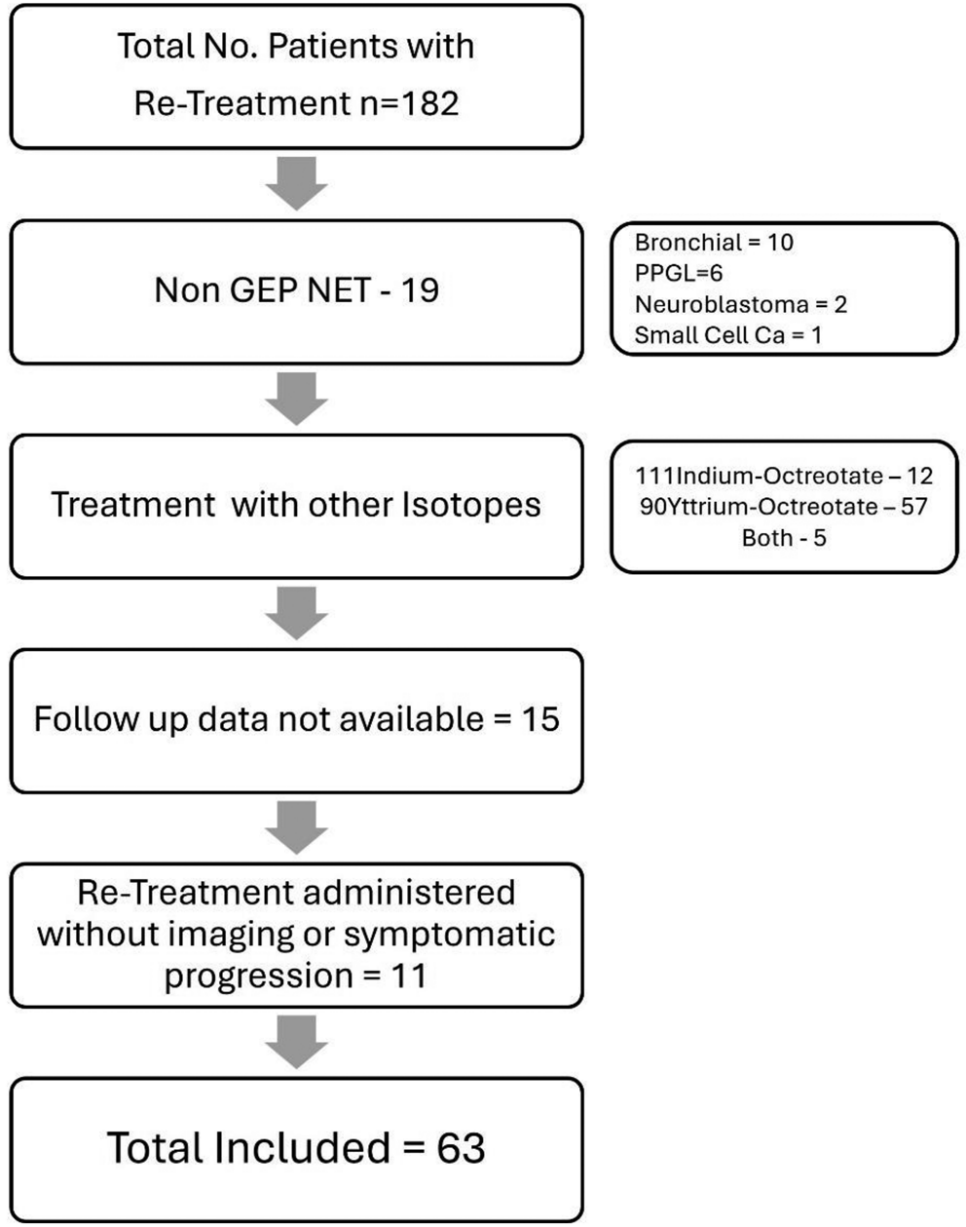
Selection of patients for the analysis

Retrospective analyses was performed for patients who were treated with LuTate at our centre over a ten-year period, between 2009 and 2019, allowing for a period of at least 24 months of follow up. Eligible patients had GEP NET of any grade who were treated with I-PRRT, and subsequently received R-PRRT due to either symptoms or imaging evidence of disease progression. Patients who received cycle/s of [^111^In] Indium-DOTA-Octreotate (In-Tate) or [^90^Y]Y-DOTA-Octreotate (Y-Tate) during initial or re-treatment were excluded. Patient selection is detailed in Fig. 1 below.

Patient demographic data, tumour histology and grade, radiopharmaceutical administered activity per cycle, total number of treatments administered, and haematological and biochemical toxicity prior to, during and 3 months post LuTate were collected. Baseline and 3 months post I-PRRT or R-PRRT [^68^Ga]Ga-DOTA-Octreotate (GaTate) PET/CT, [^18^F]Fluoro-deoxy glucose (FDG) PET/CT and structural imaging (where available) were analysed for response assessment.

### Treatment protocol

Each cycle of LuTate treatment was administered intravenously with oral premedication including 2 mg of granisetron and 8 mg of dexamethasone, to reduce potential adverse effects of nausea related to amino acid infusion and post-treatment flare of symptoms. Discharge medications include two days of granisetron (2 mg daily) and dexamethasone (4 mg daily) following treatment. Reno-protective amino acid infusion (25 g lysine and 25 g arginine in 1 L of normal saline) was commenced 30 min prior to treatment and continued for 3 h after LuTate therapy, except in patients with impaired renal function (GFR < 60 ml/min) in whom infusion was extended to 4 h [21].

Generally, 2 cycles of LuTate R-PRRT were administered at 6–10 week intervals for the retreatment protocol. Radiosensitising chemotherapy was administered if prior induction LuTate showed benefit of its use unless contra-indicated by prior toxicity or comorbidities. Capecitabine was given in 825 mg/m^2^ (bd), 2 days prior to PRRT administration and continued for 2 weeks. For the CAPTEM regimen, reserved for G2-3 pancreatic NET, especially if FDG-avid on PET/CT, capecitabine was given as 750 mg/m^2^ oral bd (max 2500 mg/day) days 1–14 commencing 2 days prior to LuTate therapy, and temozolomide 100 mg/m^2^ bd (75 mg/m^2^ if extensive prior chemotherapy) commencing one day after LuTate for 5 days. Blood tests including full blood count, and renal and liver function biochemistries were performed at 2- and 4-weeks following completion of each treatment cycle.

### Nomenclature of re-treatments

An administration of LuTate for imaging, biochemical or symptomatic progression following response to initial PRRT (I-PRRT) was termed as Re-treatment (R-PRRT) for the purpose of this study. The I-PRRT typically consisted of 4 cycles of LuTate at 8 GBq per administration, although administered activity was adapted for disease burden and renal function [22]. Each R-PRRT typically consisted of 2 cycles of LuTate at 8-12 week intervals; however, this varied between 1 and 3. Hence the first re-treatment course was termed R1-PRRT, second as R2-PRRT and so on

### Follow-up>

Post-treatment restaging PET/CT imaging was typically performed at 3 months after the last cycle of LuTate in line with current response assessment guidelines [23]. Imaging consisted of GaTate PET/CT in all patients. Structural imaging (CT and/or MRI) was analysed when available. FDG PET/CT was performed in G2 or G3 disease or if the disease was known to be FDG avid prior to R-PRRT

Objective Response Rate (ORR) was assessed using RECIST1.1 criteria when diagnostic imaging was available. In instances where structural imaging was not available, molecular imaging guided RECIST measurements were documented as described in a prior publication [24]. Response on molecular imaging including GaTate and FDG was also documented as per previously defined criteria [24].

Progression free survival (PFS) was defined from the time of R1-PRRT administration to disease progression biochemically or on imaging (defined by the composite criteria), death or commencement of the next treatment required for symptomatic control. Composite criteria for progression included one or more of (a) appearance of new lesions on molecular imaging, (b) increase in size of tracer avid lesions on PET (if non-measurable on CT), (c) radiological progression unless clearly demonstrated to represent pseudoprogression related to cystic necrosis based on low CT density and negative GaTate and FDG PET, (d) development of uncontrolled hormonal symptoms or pain on any ongoing treatment and (e) serial increase in serum chromogranin not attributable to renal or cardiac impairment. Next treatment modality is defined as any other oncologic therapy, including R-PRRT. Overall survival (OS) of the entire cohort was assessed from first cycle of I-PRRT.

Haematological and renal toxicity were assessed during LuTate and at 3 months post LuTate using CTCAE v5.0 criteria. Therapy-related myeloid neoplasms (t-MN) were documented.

Exploratory analyses were performed to identify factors associated with ORR and PFS after R1-PRRT. Factors to explore include primary site, disease grade, FDG-avidity in G2/3 NET, the total number of R-PRRT treatment courses (if more than one), use of radiosensitising chemotherapy, dominant site of metastases (liver, nodes, lung, bone, primary, multifocal) and size of largest metastatic lesion (< 2, 2–4, >4 cm).

### Statistical analysis

The disease control rate (DCR) (defined as stable disease and any partial or complete response on imaging), obtained on the same patients post I-PRRT and R1-PRRT were compared using McNemar’s method by estimating the risk difference for paired binary data [25].

For time to event analysis of PFS or OS, the Kaplan-Meier method was used to estimate survival curves using the time origin given in the endpoint definitions. For PFS, Cox regression was used to estimate hazard ratios (HRs) associated with selected explanatory variables (EVs). Logistic regression was used to examine association between selected EVs and the DCR post R1-PRRT. In all cases where association with EVs was examined, HRs or odds ratios (ORs) were estimated from unadjusted regression models. For the primary site and dominant site EVs, the reference category was all other sites combined. For all other EVs, a specific reference category was chosen.

The assumption of proportional hazards was evaluated by examining the time constancy of regression coefficients and diagnostic plots. No adjustments were made for multiple comparisons. All subgroup analyses done were stated. Statistical inference is not regarded as definitive for the following reasons: (1) the study design is an historical cohort based on an audit of routinely and retrospectively collected clinical data, (2) analysis involved decisions made post-hoc to data collection, (3) analysis involved subgroups and multiple comparisons. For these reasons, the 95% confidence intervals (CIs) reported are approximations and *P*-values have not been provided.

All statistical analyses were performed in R v4.2 or higher.

## Results

The demographic details, site of primary, and GEP NET grade (G) are detailed in Table 1. A total of 63 patients were eligible for analysis. All 63 patients had I-PRRT treatment, followed by subsequent R1-PRRT. Of these 63 patients, 20 (32%) underwent R2-PRRT and 6 (9.5%) had R3-PRRT. A primary originating from the small bowel was most common (51%) followed by a pancreatic origin (40%). The majority of patients (70%) had G2 disease.

**Table 1:** Demographic details of patients

Characteristic	*N* = 63
Sex of patient	
Female	32 (51%)
Male	31 (49%)
Age	Median (IQR): 68 (59, 74)
Site of Primary	
Pancreas	25 (40%)
Small Bowel	32 (51%)
Colon	0 (0%)
Rectum	3 (4.8%)
Unknown (but presumed small bowel due to imaging findings of mesenteric nodes or hepatic dominant disease)	3 (4.8%)
Grade of NET	
G1	8 (12%)
G2	44 (70%)
G3	6 (10%)
Unknown	5 (8%)

The indications and details of re-treatments are in Table 2.

**Table 2:** Details of lutate treatments

	Number Of patients treated	Mean cumulative dose GBq	Median cycles administered	Radio-sensitizing chemotherapy	Indication for treatment
Initial PRRT (I-PRRT)	63	28.3 (12.7–43.9)	4	45 • Capecitabine – 38 • 5FU – 4 • CAPTEM − 3	
Re-treatment 1 (R1-PRRT)	63	14.2	2	34 • Capecitabine – 29 • 5FU – 2 • CAPTEM − 3	Imaging Progression − 57 Symptom Progression- 4 Biochemical Progression – 2
Re-treatment 2 (R2-PRRT)	20	12.1	2	12 • Capecitabine – 10 • CAPTEM − 2	Imaging progression – 17 Symptom progression – 3
Re-treatment 3 (R3-PRRT)	6	13.8	1.5	4 (Capecitabine)	Imaging progression − 6

### Survival analysis

**Fig. 2:**
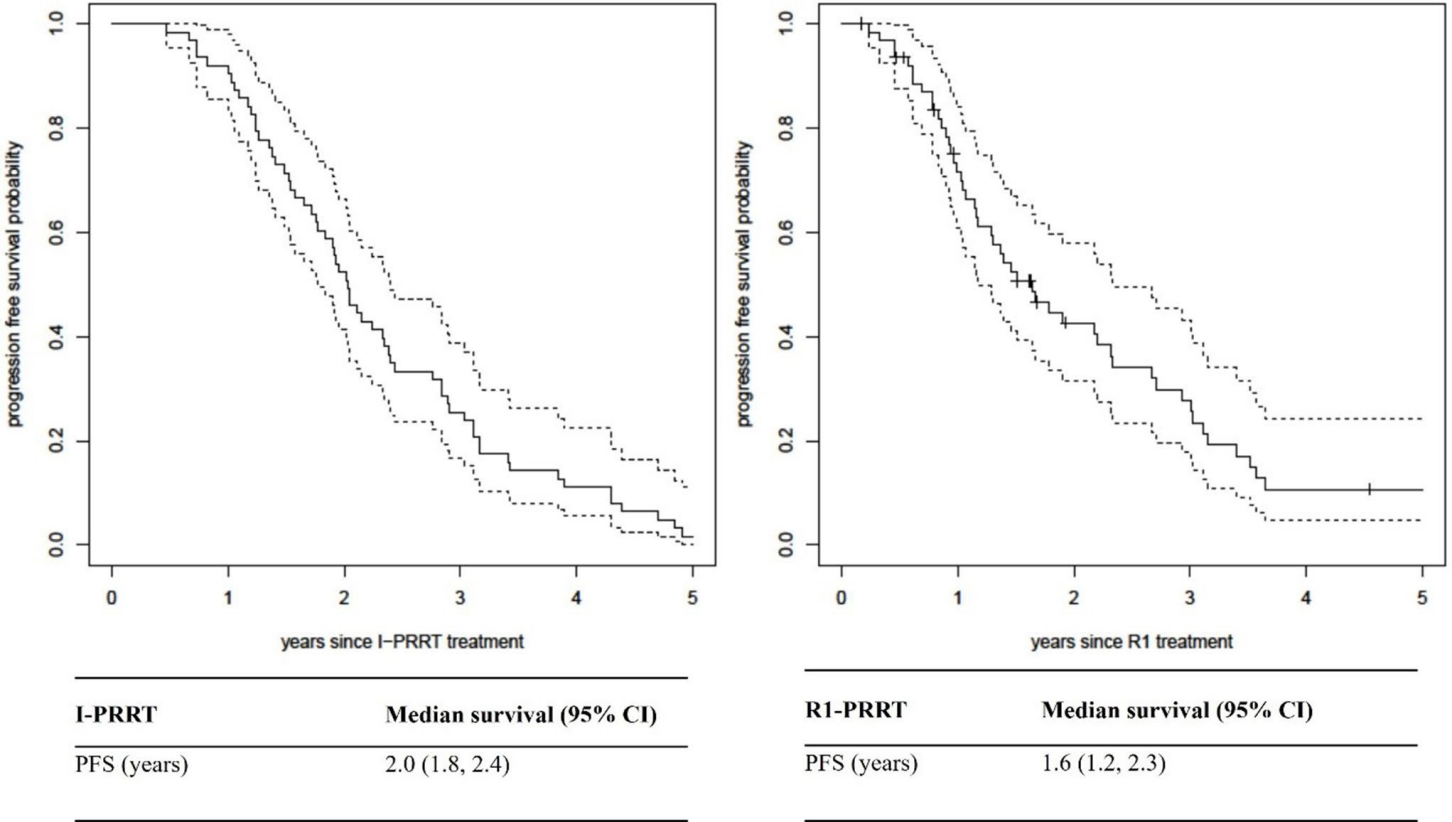
PFS median survival following I-PRRT and R1-PRRT

Median PFS following I-PRRT was 2.0 years (95% CI 1.8–2.4 years) with 92% of patients deemed not to have progressed at 1 year (Fig. 2).

The median PFS following R1-PRRT (calculated from the date of R1) was 1.6 years or 19 months (95% CI 1.2–2.3 years). Of the 63 patients, 72% patients were deemed not to have progressed at 1 year after R1-PRRT (Fig. 2).

There were 20 patients treated with R2-PRRT, of whom 6 died and 11 further progressed (at time of analysis) and 3 were on follow up with ongoing stable disease. Although the PFS probability was not estimated due to small numbers, the PFS event rate from R2-PRRT till the end of follow-up (median 1 year) was 44 cases per 100 person-years (95% CI: 27, 73).

Among the 6 patients treated with R3-PRRT, all progressed including 4 deaths during the follow up period. Owing to the small number of events, survival probabilities were not estimated. Median time to event was 8 months post R3 (range 6–20 months).

The OS of the entire cohort from the date of I-PRRT was 6 years (95% confidence interval 4.7–7.5 years) with a total of 35 deaths in the cohort.

### Toxicity

#### Renal function

There was no worsening of renal function following R1-PRRT. At 3 months post R2-PRRT, worsening GFR from CTCAE G2 to G3 was seen in 2/20, with no further GFR deterioration following R3-PRRT.

#### Haematological

Following R1-PRRT, two patients had G 3 thrombocytopenia and 1 had G4 thrombocytopenia. One patient experienced a bleeding episode due to co-existing haemorrhoids (in the context of G4 thrombocytopaenia).

Following R2-PRRT no G3 anaemia or thrombocytopenia was seen. One patient experienced G3 leukopenia.

Following R3-PRRT, G 3 thrombocytopenia occurred in 1 patient. No other G 3 haematological or renal toxicities or were seen.

Myelodysplastic syndrome (MDS) was reported in 3 of the 63 patients and acute myeloid leukemia (AML) was diagnosed in one patient. The details are in Table 3. Of note, all patients who developed MDS/AML had only mild (G1/2) haematological toxicity (G2 thrombocytopenia *n* = 1, G2 anaemia *n* = 2, G2 lymphopenia *n* = 1) post LuTate therapies. The first patient in Table 3 had persistent anaemia and a bone marrow aspirate showed myelodysplasia with no cytogenetic abnormalities. However, the white cell count and platelet count were within normal limits and this allowed for further retreatment with PRRT following discussion in our multi-disciplinary meeting.

**Table 3:** Details of patients who developed MDS/AML

	LuTate cycles	Administered activity (GBq)	Time to diagnosis of event from C1 I-PRRT	Prior chemotherapy
1	I-PRRT – 3 (with Capecitabine) R1-PRRT – 1 R2 -PRRT – 1	I PRRT – 18.9 R1-PRRT – 8.1 R2-PRRT – 7.8	MDS − 18 months (Prior to R1-PRRT)	Anthracycline chemotherapy prior to PRRT
2	I-PRRT – 4 R1-PRRT – 2	I PRRT – 35.5 R1-PRRT – 20.1	MDS − 36 months (Post R1-PRRT)	None
3	I-PRRT – 4 (with Capecitabine) R1-PRRT – 1	I PRRT – 31.5 R1-PRRT – 9.1	MDS − 23 months (Post R1-PRRT)	None
4	IPRRT – 4 (Capecitabine) R1-PRRT – 1 R2-PRRT – 1	I-PRRT − 33.2 R1-PRRT – 5.8 R2-PRRT – 6.9	AML diagnosed 10 years from C1 I-PRRT (Post R2-PRRT)	None

### Response to treatment

Response rates are detailed in Table 4 for RECIST 1.1 measurements, and Table 5 for molecular imaging. For I-PRRT, baseline imaging was available in 58/63 patients, of which RECIST measurable disease was present at baseline in 44 patients. However, 5 patients did not have subsequent post-therapy imaging at 3-months to allow for comparison. Baseline imaging prior to retreatment was available for measurements in 62/63 patients for R1-PRRT. Of the 62, measurable disease was present in 41 patients. Subsequent follow up imaging to allow RECIST measurements was not available in 20 of the 62 patients.

**Table 4:** RECIST 1.1 responses following I-PRRT and R1-PRRT

RECIST 1.1 Response	I-PRRT	R1-PRRT
Measurable	44	41
RECIST not evaluable	19	22
CR	1/44 (2.2%)	0
PR	24/44 (54.5%)	4/41 (9.7%)
SD	26/44 (59%)	32/41 (78%)
PD	2/44 (4.5%)	6/41 (14.6%)

(CR – Complete response, PR – Partial response, SD – Stable disease, PD – Progressive disease)

**Table 5:** Molecular imaging (SSTR) response rates following treatments

	I-PRRT	R1 PRRT
GaTate Response		
CR	2/60 (3.3%)	4/58 (6.9%)
PR	44/60 (73%)	27/58 (47%)
SD	13/60 (22%)	21/58 (36%)
PD	1/60 (1.7%)	6/58 (10%)
Imaging unavailable	3*	5*

(CMR- Complete metabolic response, PMR- Partial metabolic response, CR – Complete response, PR – Partial response, SD – Stable disease, PD – Progressive disease, * - Imaging was not available either due to being done at other centres or abandoned due to disease progression. [^111^In] Indium- pentetreotide (Octreoscan) imaging was done as baseline imaging in some patients and hence could not be used for comparison with post therapy GaTate, which was introduced in our facility in 2009, but only gradually replaced Octreoscan imaging in patients previously followed with this agent)

For RECIST 1.1 response, disease control was achieved in approximately 96% of patients with measurable disease following I-PRRT. After R1-PRRT, RECIST 1.1 DCR was seen in approximately 85.5% of patients with measurable disease.

**Fig. 3:**
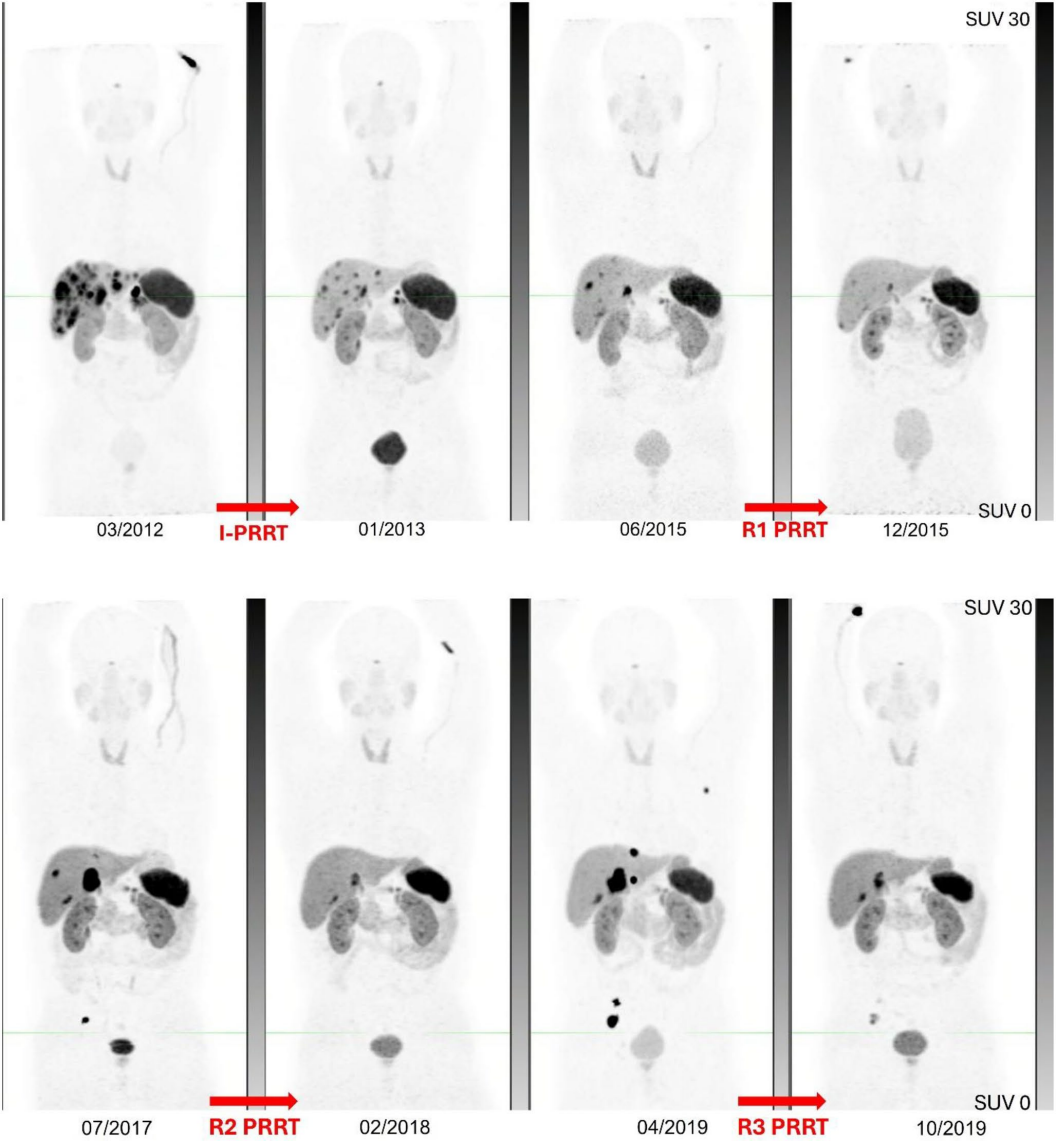
Legend: A 62 year male with metastatic pNET G2 at diagnosis progressed to G3, had undergone chemotherapy with Carboplatin / Etoposide prior to I-PRRT. He underwent R-PRRT treatments (shown up to R3 in the images above) for imaging progression on several occasions with good partial response to each treatment. A total of 5 R-PRRT treatments were administered before the disease was deemed radio-resistant and further second line chemotherapy (CAPTEM) was administered almost 12 years after the I-PRRT.

GaTate response was assessed following I-PRRT and R1-PRRT treatment occasions in 57/63 (90%) patients. GaTate disease control rates were 56/57 (98%) after I-PRRT and 51/57 (89%) after R1-PRRT. Figure 3 demonstrates GaTate response in a patient who underwent multiple re-treatments.

FDG and RECIST disease control rates were not compared in this way owing to missing data.

### Response in G3 GEPNET

A total of 6 patients had a G3 NET with 3 of pancreatic origin, 2 of small bowel origin and 1 rectal primary. Following I-PRRT, 4/6 showed partial response on molecular imaging while 2/6 had stable disease on molecular imaging. Half the patients showed RECIST PR, and the other half had stable disease following I-PRRT on RECIST criteria. Of these, 3 patients had both R1 and R2-PRRT cycles. Following R1-PRRT, 3 showed complete response, 2 partial response and 1 had stable disease. RECIST responses were non-evaluable in 3/6 post R1-PRRT. Overall, there was a good molecular imaging response following both I-PRRT and R1-PRRT in G3 patients.

### Association between clinical factors and PFS

Patients with primary tumor of small bowel origin (odds ratio 6.3, CI 0.93–125) and G2 disease (OR 4.3, CI: 0.51-29.0) showed better disease control on GaTate molecular imaging. Other factors were not associated with response on GaTate PET/CT. Details are shown in supplementary material Table 7.

In 18/63 patients a different line of treatment was subsequently used upon further progression (when clinical status or disease phenotype on functional imaging favoured such treatments over R-PRRT). These treatments included chemotherapy, everolimus, hepatic embolisation or external beam radiotherapy.

## Discussion

This is a retrospective study to evaluate the efficacy and outcome of LuTate re-treatment for patients with progressive GEPNET, from an ENETS Center of Excellence. R-PRRT was administered based on imaging, biochemical or symptoms progression after initial responses to I-PRRT. Accordingly, they represent a subgroup of patients who were already deemed to have benefitted from PRRT without limiting toxicity. They were also preselected by having a favourable imaging phenotype based on SSTR imaging and FDG PET/CT when indicated by tumour grade or prior imaging.

Several other studies have reported on the efficacy of re-treatment peptide receptor radionuclide therapy (PRRT) upon disease progression [11–19]. The study by Sistani et al. [20] however, administered maintenance doses of PRRT 6 monthly for up to 4 years.

All 63 patients in this study received at least 1 cycle of R1-PRRT. R2-PRRT was administered in 20 patients and 6 patients underwent R3-PRRT. In our series, the median PFS of R1-PRRT was median of 19 months (1.6 years). This is similar to the results from other studies [11, 12, 15–18]. The durability of response appears slightly lower than the initial treatment course, where the median PFS following I-PRRT was 2 years. The PFS benefit is still considered clinically meaningful in the context of progressive disease and especially as the majority of patients had G2 disease. There appears to be a trend in shortening of the PFS following each LuTate re-treatment similar to other prior studies. This could possibly be explained by selective progression of radio-resistant tumour clones. Of note, the median overall survival of the entire cohort from the I-PRRT of 6 years is encouraging, suggesting LuTate is an effective therapy for patients with SSTR expressing NET despite progression after I-PRRT.

There was an overall favourable objective disease control rate with PRRT re-treatment. On GaTate PET, the disease control rate was close to 90% and based on molecular imaging guided RECIST, the disease control was achieved in up to 85% of the patients. One of the limitations of our study is that due to the retrospective nature, the FDG PET/CT and diagnostic structural imaging data suitable for RECIST analysis was not available for all patients.

In our series, re-treatment with LuTate was tolerable. There was no worsening of renal function following R1-PRRT administrations. Renal toxicity had been reported in some prior studies [11, 15, 18]. The most common G3 or 4 haematological toxicity was overall low: thrombocytopenia in 4 patients overall. On longer term follow-up, MDS was detected in 3 patients, and AML in 1 patient (~ 6% of the cohort). The series by van der Zwan et al. [12] documented 2 cases of MDS and 2 cases of AML following R-PRRT (2.2% of the cohort). In our series, radio-sensitising chemotherapy was used in 50 of the 63 patients for I-PRRT and 35/63 patients with R1-PRRT. Whether this has contributed to MDS/AML above LuTate alone rate is unknown. The overall mechanism of t-MN is poorly understood, and further prospective evaluation is warranted.

Existing literature is varied with the isotopes used. Zemczak et al. [18] used tandem administration of both Ytate and LuTate. Patients in the series by Vaughan et al. [15] had either 90-Yttrium or 177-Lutetium based therapy with 90-Y PRRT administered intra-arterially in some patients with liver predominant disease. In contrast to this, we analysed only patients who received one form of treatment which is LuTate for both I-PRRT and R-PRRT.

The study by Van der Zwan [12] had the largest cohort of patients using LuTate re-treatment and they included a control arm of patients who did not have re-treatments, and demonstrated a statistically significant prolongation of OS in the group with retreatment-PRRT. They however included patients only of G1 and G2 disease and also included non-GEP NET in the cohort.

Our data adds to the existing literature on the efficacy of LuTate re-treatment. The strengths of our data are the single radionuclide inclusion (LuTate only) as well as GEP NET inclusion (excluding bronchial NET and pheochromocytoma/paragangliomas) although this constitutes a heterogeneous group. Ours is one of the larger series in terms of the number of patients studied with long survival follow-up. In our study, while 63 patients underwent both I-PRRT and R1-PRRT, the number who underwent further R-PRRT is much smaller to draw any meaningful conclusions on survival data on R2- and R3- PRRT. We anticipated a decreasing efficacy of R-PRRT with subsequent re-treatment cycles but the small number of patients precluded statistical analysis. The current results are reflective of GEPNETs originating from different organs with different grades. The limited number of patients in each subgroup was again an impediment to detailed subgroup (by primary or grade) analysis of these outcomes. Other limitations of our study include the retrospective nature, the lack of comparative arm, the variable use of radiosensitising chemotherapy, inconsistencies and missing data and hence inability to draw definite conclusions in certain aspects such as estimation of difference in response rates with each re-treatment and analysis of prognostic and predictive factors. It is difficult to ascertain the effects of radiosensitising chemotherapy with response or t-MN in this series [26], but our results did not suggest longer PFS with radiosensitizing chemotherapy. However, given the overall favourable responses and outcome similar to prior studies, further prospective multi-centre trial comparing R-PRRT with second line systemic treatments is warranted. The NET RETREAT Trial comparing [^177^Lu]Lu-DOTA-TATE re-treatment vs. Everolimus in patients with metastatic midgut NET is currently in progress (NCT05773274).

## Conclusion

For patients with progressive GEP NET with response to initial PRRT, re-treatment LuTate is well tolerated achieving durable disease control and favourable overall survival. Careful selection of patients in a multidisciplinary setting to maximise benefit of re-treatment balancing potential longer-term toxicity should be considered.

## Electronic supplementary material

Below is the link to the electronic supplementary material.

Supplementary Material 1

## Data Availability

Individual participant data that underlie the results reported in this publication, after deidentification (text, tables, figures and appendices) and the study protocol will be available beginning 6 months and ending 36 months after publication to researchers whose proposed use of the data has been approved by an independent review committee identified for individual participant data metaanalysis. Proposals may be submitted up to 36 months following article publication. After 36 months, the data will be available at the institutional data warehouse but without investigator support other than deposited metadata. Information regarding submitting proposals and accessing data may be directed to DGO@petermac.org.
