# Supplementary material for: Safety and efficacy of re-treatment with [177Lu]Lu-DOTA-Octreotate radionuclide therapy in progressive gastro-entero-pancreatic neuroendocrine tumours – a single centre experience
Source: Eur J Nucl Med Mol Imaging. 2025 Mar 26;52(10):3672–81. doi: 10.1007/s00259-025-07235-w (PMC12316713; doi:10.1007/s00259-025-07235-w)
Supplement: Supplementary file 1 — Supplementary Material 1 [file 259_2025_7235_MOESM1_ESM.docx]

Supplementary material

Supplementary Table 7. **Unadjusted association with GaTate response at R1**

| **Characteristic** | **N** | **OR**^1^ | **95% CI**^1^ |
| --- | --- | --- | --- |
| age at R1 (years) | 58 | 0.98 | 0.90, 1.05 |
| sex of patient | 58 |  |  |
| female |  | 1.00 | — |
| male |  | 0.16 | 0.01, 1.07 |
| primary site: pancreas | 58 | 0.12 | 0.01, 0.78 |
| primary site: small bowel | 58 | 6.30 | 0.93, 125 |
| grade of disease | 54 |  |  |
| grade 1 |  | 1.00 | — |
| grade > = 2 |  | 4.30 | 0.51, 29.0 |
| was bone a dominant site of disease | 58 |  |  |
| no |  | 1.00 | — |
| yes |  | 0.42 | 0.07, 3.34 |
| Radio sensitizing Chemotherapy | 58 |  |  |
| Yes |  | 1.00 | — |
| No |  | 0.68 | 0.12, 3.97 |
| ^1^OR = odds ratio, CI = confidence interval | | | |

Table 8 A**ssociation with PFS from R1**

| **Characteristic** | **N** | **HR**^1^ | **95% CI**^1^ |
| --- | --- | --- | --- |
| age > 60 years | 63 | 1.33 | 0.72, 2.48 |
| sex of patient | 63 |  |  |
| female |  | 1.00 | — |
| male |  | 1.43 | 0.82, 2.49 |
| primary site: pancreas | 63 | 1.22 | 0.70, 2.14 |
| primary site: small bowel | 63 | 0.61 | 0.35, 1.08 |
| grade of disease | 58 |  |  |
| grade 1 |  | 1.00 | — |
| grade > = 2 |  | 0.66 | 0.29, 1.49 |
| was bone a dominant site of disease | 63 |  |  |
| no |  | 1.00 | — |
| yes |  | 2.47 | 1.25, 4.86 |
| Radio sensitizing Chemotherapy | 63 |  |  |
| Yes |  | 1.00 | — |
| No |  | 1.51 | 0.83, 2.76 |
| ^1^HR = hazard ratio, CI = confidence interval | | | |
